# Testing the Feasibility of Sensor-Based Home Health Monitoring (TEC4Home) to Support the Convalescence of Patients With Heart Failure: Pre–Post Study

**DOI:** 10.2196/24509

**Published:** 2021-06-03

**Authors:** Kendall Ho, Helen Novak Lauscher, Jennifer Cordeiro, Nathaniel Hawkins, Frank Scheuermeyer, Craig Mitton, Hubert Wong, Colleen McGavin, Dianne Ross, Glory Apantaku, Mohammad Ehsan Karim, Amrit Bhullar, Riyad Abu-Laban, Suzanne Nixon, Tyler Smith

**Affiliations:** 1 Digital Emergency Medicine University of British Columbia Vancouver, BC Canada; 2 University of British Columbia Vancouver, BC Canada; 3 Centre for Clinical Epidemiology & Evaluation University of British Columbia Vancouver, BC Canada; 4 Centre for Health Evaluation & Outcome Sciences University of British Columbia Vancouver, BC Canada; 5 BC Support for People & Patient-Oriented Research & Trials Vancouver, BC Canada; 6 Vancouver General Hospital Vancouver, BC Canada; 7 TELUS Health Vancouver, BC Canada

**Keywords:** telemonitoring, heart failure, home health monitoring, technology, telehealth, emergency care, community care, emergency department, quality of life, self-efficacy

## Abstract

**Background:**

Patients with heart failure (HF) can be affected by disabling symptoms and low quality of life. Furthermore, they may frequently need to visit the emergency department or be hospitalized due to their condition deteriorating. Home telemonitoring can play a role in tracking symptoms, reducing hospital visits, and improving quality of life.

**Objective:**

Our objective was to conduct a feasibility study of a home health monitoring (HHM) solution for patients with HF in British Columbia, Canada, to prepare for conducting a randomized controlled trial.

**Methods:**

Patients with HF were recruited from 3 urban hospitals and provided with HHM technology for 60 days of monitoring postdischarge. Participants were asked to monitor their weight, blood pressure, and heart rate and to answer symptomology questions via Bluetooth sensors and a tablet computer each day. A monitoring nurse received this data and monitored the patient’s condition. In our evaluation, the primary outcome was the combination of unscheduled emergency department revisits of discharged participants or death within 90 days. Secondary outcomes included 90-day hospital readmissions, patient quality of life (as measured by Veterans Rand 12-Item Health Survey and Kansas City Cardiomyopathy Scale), self-efficacy (as measured by European Heart Failure Self-Care Behaviour Scale 9), end-user experience, and health system cost-effectiveness including cost reduction and hospital bed capacity. In this feasibility study, we also tested the recruitment strategy, clinical protocols, evaluation framework, and data collection methods.

**Results:**

Seventy participants were enrolled into this trial. Participant engagement to monitoring was measured at 94% (N=70; ie, data submitted 56/60 days on average). Our evaluation framework allowed us to collect sound data, which also showed encouraging trends: a 79% reduction of emergency department revisits post monitoring, an 87% reduction in hospital readmissions, and a 60% reduction in the median hospital length of stay (n=36). Cost of hospitalization for participants decreased by 71%, and emergency department visit costs decreased by 58% (n=30). Overall health system costs for our participants showed a 56% reduction post monitoring (n=30). HF-specific quality of life (Kansas City Cardiomyopathy Scale) scores showed a significant increase of 101% (n=35) post monitoring (*P*<.001). General quality of life (Veterans Rand 12-Item Health Survey) improved by 19% (n=35) on the mental component score (*P*<.001) and 19% (n=35) on the physical component score (*P*=.02). Self-efficacy improved by 6% (n=35). Interviews with participants revealed that they were satisfied overall with the monitoring program and its usability, and participants reported being more engaged, educated, and involved in their self-management.

**Conclusions:**

Results from this small-sample feasibility study suggested that our HHM intervention can be beneficial in supporting patients post discharge. Additionally, key insights from the trial allowed us to refine our methods and procedures, such as shifting our recruitment methods to in-patient wards and increasing our scope of data collection. Although these findings are promising, a more rigorous trial design is required to test the true efficacy of the intervention. The results from this feasibility trial will inform our next step as we proceed with a randomized controlled trial across British Columbia.

**Trial Registration:**

ClinicalTrials.gov NCT03439384; https://clinicaltrials.gov/ct2/show/NCT03439384

## Introduction

Heart failure (HF) is common, life-limiting, and the leading cause of hospitalization in North America and Europe [1]. Patients with HF are affected by debilitating symptoms and impaired quality of life. The median survival after hospitalization is 2.5 years, while rehospitalization rates exceed 20% at 30 days [1]. Telemonitoring has been proposed to support patients with HF in the community. Clinical studies to date have demonstrated promising but inconsistent evidence for this strategy. A recent meta-analysis of 17 studies of telemonitoring found mixed results with benefits reported in some areas and no significant change in others [2]. Two large randomized controlled trials (RCTs) failed to find a reduction in death or hospitalization [3,4]. These studies did not examine issues such as patients’ level of familiarity and adoption of these technologies [3], or integration of technology-enabled monitoring in the context of convalescent care [4]. The Telemedical Interventional Management in Heart Failure II (TIM-HF2) trial demonstrated significant reductions in all-cause mortality and days lost due to unplanned cardiovascular hospitalization 1 year after monitoring [4]. Further, a meta-analysis concluded that a subpopulation of patients with HF recently discharged from hospital within 28 days of admission benefited preferentially from home telemonitoring and showed reduced mortality and all-cause hospitalizations [5]. Further trials of home telemonitoring for patients with HF are required to clarify these findings and their underlying factors.

Telehealth for Emergency-Community Continuity of Care Connectivity via Home Telemonitoring (TEC4Home) is a 4-year research initiative to evaluate the efficacy of home health monitoring (HHM) as an integrated component of health care delivery to support the transition of patients with HF from hospital to home. The initiative consists of a feasibility study followed by a pragmatic, multicenter, RCT [6]. The purpose of this paper is to report the results of the feasibility study conducted in preparation for the RCT, to test the clinical and technical monitoring protocols in hospital and community settings, and to refine the evaluation framework. We hypothesized that the study procedures would be acceptable to both patients and providers; that the evaluation framework would capture useful metrics for outcome evaluation; and that the results would suggest a trend toward decreasing 90-day emergency department (ED) revisits and hospital admission rates, enhancing quality of life, improving self-efficacy, and reducing health care costs for patients and the health care system.

## Methods

### Recruitment Procedures

The feasibility study was an unblinded trial. Participants were prospectively recruited from EDs and inpatient units at 3 large urban hospital sites in British Columbia from November 2016 to July 2017. As this study was designed to assess the feasibility of the study protocol and refine procedures with a purposive sample gleaned from the feasibility study sites, no power calculation was conducted. Rather, the sample size was estimated from the recruitable number of patients with HF presenting at the ED sites based on administrative data with HF diagnosis and factoring in estimation of eligibility and attrition rates.

The inclusion criteria were as follows: one or more typical clinical HF symptoms; one or more typical clinical HF signs; one or more objective measures of HF, such as brain natriuretic peptide elevation or chest x-ray findings; currently receiving diuretic therapy; and age of 19 years or above. The exclusion criteria were as follows: inability to complete study procedures; no access to a reliable phone for communicating with a nurse; coronary or structural heart intervention during admission that would alter the course of HF convalescence with medical therapies alone; patients not expecting to present back to hospital for further deterioration, such as those wanting to die at home (see Multimedia Appendix 1 for the patient participant eligibility criteria of the feasibility).

Potential participants were first identified via referrals from hospital ED staff and screening hospital ED lists. Throughout recruitment, the study team expanded referral streams to include hospital in-patient wards to increase recruitment numbers. Eligible patients were contacted in the hospital when possible or immediately after discharge. Participants completed the consent process and were enrolled within 7 days from hospital discharge.

This study obtained ethics and associated approvals (no. H16-01076) from the regions where the 3 hospitals operate: University of British Columbia (UBC), Vancouver Coastal Health, and Interior Health Authorities.

### HHM Intervention

The HHM device kits were provided by TELUS Health (technology partner). The kit included a touch screen tablet computer and Bluetooth-connected sensors that included a blood pressure cuff, a pulse oximeter, and a weighing scale. Upon enrollment and after patients returned home, monitoring nurses aimed to contact all participants within 1-3 days by telephone. Nurses trained to provide monitoring during the study period were seconded from cardiac clinics and had extensive experience managing patients with HF. During the first call, the nurse confirmed eligibility and scheduled the delivery of the HHM device kit. Kits were provided free of charge for 60 days and were delivered and set up in participants’ homes by a technician. The protocol sought to have kits delivered within 7 days of discharge from the hospital; however, this varied depending on the individual patient’s scheduling preference.

Participants were taught how to use the device kit by the technicians at the time of delivery and were observed submitting their first set of biometric measurements (ie, blood pressure, oxygen saturation, pulse, and weight) and answering 10-12 yes-or-no questions regarding symptoms (eg, “I feel more short of breath today”) in the technician’s presence. Participants were then asked to continue daily submissions of 1 set of this data per day for the next 60 days. The monitoring nurse sent an enrollment letter to the participant’s primary care provider (PCP) and other health care team members (eg, specialists), if appropriate, to notify them of their patients’ participation in the monitoring program and research study. PCPs or specialists were also asked to review and change the default monitoring limits of the patients (eg, blood pressure, heart rate limits, or oxygen saturation levels) to customize to patients’ needs if required. Dry target weight was also requested if known.

Monitoring data were reviewed by a single monitoring nurse Monday through Friday between 8:00 AM and 4:00 PM (excluding statutory holidays) via a web-based dashboard. The primary monitoring nurse performing the monitoring was a cardiac specialty nurse with over 20 years of experience working in cardiac care settings, such as a heart function clinic.

The platform listed and flagged patient measurements falling outside of predefined default (or customized) values (eg, weight gain of more than 5 lbs in 2 days), changes in symptoms, or missed data submissions. Alerts were flagged as red/severe (eg, systolic blood pressure < 85 mm Hg or >160 mm Hg) or yellow/caution (eg, systolic blood pressure 85-89 mm Hg or 141-160 mm Hg). The monitoring nurse followed up by telephone with the participants on all concerning flags and alerts. The monitoring nurse was authorized to advise the patient on better managing their condition and to provide education on management of the patient’s condition. For medication changes, the monitoring nurse would connect with the most responsible physician (eg, PCP) of the patient to facilitate these changes. In addition, the monitoring nurse would connect with other specialists with whom the patient was attached, if needed. For urgent situations, such as severe shortness of breath, the monitoring nurse was advised to direct the patient to their closest urgent care center or to call 911. Interventions that resulted from consultations between patients and monitoring nurses were documented in monitoring nurses’ notes, and summaries were shared via fax with the participants’ PCP every 2 weeks or as needed. The nurse also provided HF self-management education including advance care planning discussions to participants over the telephone, referring to a binder of HF self-management materials provided to participants upon enrollment into the study. Participants were discharged from the monitoring program after 60 days with a final report summarizing the monitoring data sent to the PCP. Participants were contacted again 30 days after discharge from the HHM program by telephone to complete a follow-up survey and to obtain their feedback on the HHM monitoring program.

### Primary and Secondary Outcomes

Key metrics were drawn from the Triple Aim Plus framework [7], and both quantitative and qualitative data were collected from patient participants, participants’ PCPs, and the monitoring nurses. The primary outcome was the combination of unscheduled ED revisits in discharged participants within 90 days of discharge or all-cause death. Secondary outcomes included hospital readmission, patient participant health status (eg, quality of life, self-efficacy), end-user experience, and health system cost-effectiveness (including cost reduction and hospital bed capacity).

### Data Collection

#### Patient Participant Experience and Outcomes

Administrative data and data regarding patient participant experience and 90-day outcomes were collected via presurveys (at the time of consent) and postsurveys (30 days after monitoring discharge), with participants serving as their own controls.

Participants completed presurveys at enrollment and postsurveys from home via mail or over the telephone. The surveys, as outlined below, comprised multiple validated scales to assess pre-to-post changes in quality of life, self-efficacy, and health care utilization.

The Veterans Rand 12-Item Health Survey (VR-12) was included to measure health-related quality of life (general) [8,9]. The scale assesses 8 domains of health to produce a physical component and a mental component score.

The Kansas City Cardiomyopathy Scale (KCCQ-12) measures HF-specific quality of life [10]. The shortened 12-item scale assesses “patient-reported symptoms, function and quality of life for patients with heart failure.” Agreement between the KCCQ-12 and the full KCCQ 23-item scale, which it is derived from, has previously been tested and results in a construct validity of 0.93-0.96 in quality of life scores, 0.97 in physical limitation scores, 0.98 in social limitation scores, and 0.98-0.99 in summary scores. Test–retest reliability calculations of KCCQ-12 have resulted in scores ranging from a 0.76 to 0.91 correlation across all domains. Scoring is calculated on a 100-point scale, with a higher score indicating a better overall health status.

The European Heart Failure Self-Care Behaviour Scale 9 (EHFScBs-9) was employed to assess self-care self-efficacy [11]. Internal reliability of this scale has been previously tested with a test score of 0.80 [11]. This scale consists of a 9 to 45-point scale, with lower scores indicating better self-care. For this trial, the method of standardizing the score to a 0-100 scale was used to make interpretation easier [12].

The My Healthcare Utilization Survey was included, as it assesses health care resource utilization and can collect information about the type and frequency of health-related services used within a specified time period. This scale was developed for this trial in collaboration with the UBC School of Population and Public Health

The System Usability Scale (postsurvey only) was also used, as it can evaluate the usability of a technology-based application [13]. The scale is scored on a 0 to 100-point scale, with higher scores representing better usability.

Basic demographic data (presurvey only) were also collected from participants and included age, sex, education level, and ethnicity.

Optional interviews were offered to all patient participants who completed the 90-day follow-up survey to gather additional feedback about their overall experience.

#### Health Care Provider Experience

After study completion, the monitoring nurses were interviewed to explore their overall experience with the monitoring model, the benefits and challenges, and suggestions for improvement (see Multimedia Appendix 2 for the monitoring nurse interview protocol).

Participants’ PCPs were invited to provide feedback regarding the HHM intervention’s impact on care delivery and workload through surveys, which were faxed to them (see Multimedia Appendix 3 for the primary care physician survey protocol).

### Statistical Analysis

#### Patient Participant Experience and Outcomes

Survey data were entered into a Research Electronic Data Capture (REDCap) database [14,15] hosted at the Centre for Health Evaluation & Outcome Sciences (CHEOS) and were analyzed using R statistical package version 3.5.3 (R Foundation for Statistical Computing). The pre-to-postsurveys were coded and scored according to the validated scales’ instructions, and paired *t* tests were used to assess pre-to-post difference across the outcome measures. The results are described as means with SD for parametric data and medians with IQRs for nonparametric data.

Pre–post analysis of administrative data was performed using Microsoft Excel (Microsoft Corporation). To determine pre–post change, we calculated the absolute risk reduction. Pre- and posthealth care costs and impacts were calculated using the self-reported health care utilization surveys. To calculate out-of-pocket costs, we used participants’ self-reported data on expenses related to their health condition, which included information on drugs, aids to daily living, housekeeping or home care, and transportation to and from medical appointments.

Interviews with patient participants about the overall experience were recorded and transcribed verbatim. Transcripts were coded, and content analysis was performed to summarize the interviews into themes (see Multimedia Appendix 4 for the patient participant interview analysis codebook).

#### Health Care Provider Experience

Survey and interview data collected from the monitoring nurses and the PCPs were summarized using content analysis to draw out themes and recommendations to guide future HHM implementations. Interview data were recorded and transcribed verbatim. Transcripts were coded and summarized by 2 researchers (AB and an undergraduate student) into main themes, including level of satisfaction for providing care, communication with participants, impact, and areas for improvement (see Multimedia Appendix 5 for the monitoring nurse interview analysis codebook).

## Results

### Recruitment

From October 2016 to June 2017, 519 patients who met the criterion of presenting to the hospital with shortness of breath were referred and screened for further HF eligibility screening: 219 met the study eligibility criteria for HF, and 70 were enrolled. Out of these 70, 47 (67%) participants completed the enrollment survey and thus provided demographic information. The median age of these participants was 75 years (range: 44-93 years), 24/47 (51%) participants identified as male, 36/47 (76%) identified as White (of European descent), and 45/47 (96%) identified English as their language of preference for health care matters.

The top 3 reasons for nonparticipation included patients declining, patients not meeting clinical eligibility criteria, and inability to provide informed consent (see Multimedia Appendix 6 for a summary of the most common reasons for patients not participating). Of the 121 patients who declined to participate, most did not provide a reason (see Multimedia Appendix 7 for a summary of reasons provided by eligible patients who declined to participate).

Of the 70 participants, 47 completed and returned the enrollment survey, 49 completed and returned the 90-day survey, and 35 completed and returned both the enrollment and 90-day survey. The subsamples providing data for each area of analysis are specified in the following section.

### HHM Adherence

Participants were expected to be in the monitoring system for 60 days, submitting data once per day. Actual monitoring adherence (ie, the actual days data were entered) averaged 56 days across our sample, which is a 94% adherence rate based on the 60 days of baseline expectation.

Satisfaction with the monitoring platform as measured by the System Usability Scale [13] resulted in a mean score of 80.0 and a median of 81.4 on a 100-point scale (n=49).

### Patient Participant Experience and Outcomes

#### Health Care Utilization

Administrative data were available for 2 of the 3 participating sites that included 36 of the 70 participants (51%). ED visits, rehospitalizations, and length of stay all decreased for these participants. Furthermore, the overall duration of rehospitalization decreased (see Table 1).

There was a 71% reduction in hospitalization costs (**P*<.001*), along with a 58% reduction in ED visit costs, although the latter did not reach statistical significance. PCP and specialist costs were similar in the pre–post analysis.

For the 30 participants (43%) who completed survey items included in the cost analysis, we estimated a 49% reduction in out-of-pocket costs (Table 2).

**Table 1:** Administrative data of change in ED visits, hospital admissions, and length of stay (n=36).

Data type	90 days pre-TEC4Home^a^	90 days post-TEC4Home	Pre–post change (%)
ED^b^ visits	66	14	–79
Hospital admissions	54	7	–87
Length of stay (days), total (median)	440 (5.0)	71 (8.0)	–84 (60)

^a^TEC4Home: Telehealth for Emergency-Community Continuity of Care Connectivity via Home Telemonitoring.

^b^ED: emergency department.

**Table 2:** Per-patient aggregate health care utilization cost.

Cost type	Mean pre-TEC4home^a^ cost (US$)	Mean post-TEC4home cost (US$)	Cost reduction (US$), n (95% CI)	Pre–post change (%)
ED^b^ visits cost^c^	618	262	–376 (–87 to 799)	–58
PCP^d^ visits cost^e^	126	129	3 (–52 to 47)	2
LoS^f^ cost (ie, overall hospital cost)^g^	10,792	3091	–7701 (–3772 to 11,631)	–71
Specialist visits^h^	160	132	–28 (–72 to 128)	–18
Patient-reported out-of-pocket cost	357	185	–175 (–49 to –395)	–49

^a^TEC4Home: Telehealth for Emergency-Community Continuity of Care Connectivity via Home Telemonitoring.

^b^ED: emergency department.

^c^Costs calculated based on standard outpatient cost from the Canadian Institute for Health Information: US $314.15.

^d^PCP: primary care provider.

^e^Standard PCP visits cost obtained from the Ministry of Health Medical Services Commission payment schedule.

^f^LoS: length of stay.

^g^Per diem ward (1 night in hospital) from the Canadian Institute for Health Information: US $1520.20.

^h^Special visits cost obtained from the Ministry of Health Medical Services Commission payment schedule.

#### Participant Experience and Outcomes

For the 35 participants who reported a complete set of pre- and postoutcomes, disease-specific quality of life demonstrated the greatest improvement, and mental and physical general quality of life also significantly improved (Table 3). No significant change occurred in HF self-care behavior.

**Table 3:** Patient-reported outcomes pre- and post-TEC4Home.

Patient-reported outcome	Pre-TEC4Home^a^ (score out of 100)	Post-TEC4Home (score out of 100)	Pre–post change, mean (95% CI)	Pre–post change (%)	*P* value
Heart failure–specific quality of life (KCCQ-12^b^)	33.4	67.1	33.7 (40.05-23.84)	100.8	<.001
Health-related quality of life (VR-12^c^ mental component)	43.1	51.4	8.3(12.48-4.08)	19.2	<.001
Health-related quality of life (VR-12 physical component)	26.7	31.7	5.0 (0.83-8.12)	18.7	.02
Heart failure self-care behavior (EHFScBs-9^d^)	70.2	74.3	4.1 (14.59-3.48)	5.84	.22

^a^TEC4Home: Telehealth for Emergency-Community Continuity of Care Connectivity via Home Telemonitoring.

^b^KCCQ-12: Kansas City Cardiomyopathy Scale.

^c^VR-12: Veterans Rand 12-Item Health Survey.

^d^EHFScBs-9: European Heart Failure Self-Care Behaviour Scale 9.

#### Participant Experience

Participants who completed the postsurvey were invited to take part in a telephone interview to discuss their experiences further: 11 out of the 49 participants (22%) who completed the postsurvey participated in telephone interviews to further discuss their experiences in the study. The findings are summarized in Textbox 1. Most notably, they described TEC4Home as contributing to a sense of safety and security after the transition from the hospital:

I wasn't afraid to come home [from the hospital]…it actually brought me a lot of comfort and security once I came home.

Participants also expressed that they felt more involved in their own care as a result of participating in the home monitoring intervention:

The TEC4Home program taught me how valuable it is

to monitor my condition

**Summarized findings from patient interview feedback by themes.**

**Project satisfaction**

- Overall, all (11/11) participants described being pleased with their participation in the project.

**Experience**

- Most (9/11) reported ease of use with the technology and training provided.
- Most (8/11) expressed they were content with the support and education provided by the monitoring nurse.
- All (11/11) expressed feeling more involved in their own care.

**Perceived impact**

- A few (3/11) participants expressed that they perceived that their primary care provider appreciated the regular patient reports (most participants felt that the program did not impact the relationship, positively or negatively, with their primary care provider in any significant way).

**Challenges**

- A few (3/11) participants expressed some technical difficulties with the equipment (eg, blood pressure cuff ripped, blood pressure cuff not fitting properly, oximeter not working, weight scale not accurate).
- Some (4/11) participants wished the monitoring was a 7-day support service.

### Health Care Provider Experience

Interviews were also conducted with the 2 monitoring nurses and covered satisfaction, patient–care provider interactions, procedures and usability, and perceived impact. Findings are summarized in Textbox 2. Monitoring nurses emphasized that they perceived positive impacts on participants quality of life and confidence:

Patients were definitely more engaged with their self-management. They’re able to report changes in their symptoms and their weights and things like that.

PCPs were invited to participate in surveys, but we did not receive any responses from them.

**Summarized findings from monitoring nurse feedback by theme. Monitoring nurse feedback (n=2).**

**Satisfaction**

- Both (2/2) monitoring nurses expressed satisfaction with the project and their experiences working with patient participants.

**Patient–care provider interactions**

- Monitoring nurses expressed satisfaction with the level of care they were able to provide through the program.
- The nurses described how some primary care providers were more responsive than others but that the overall coordination of care improved because of her communications with nurse practitioners at the HF clinics, home care clinicians, and specialist physicians.

**Procedures and usability**

- The remote patient monitoring clinician station interface could have been more streamlined.
- Graphical visualizations of patients’ biometric data were mentioned as an area of improvement.
- Consenting of patients too early led to dropout or ineligibility issues later on.
- For the HF protocol, questions could be modified to understand the patient’s condition relative to the previous day (eg, “Is your shortness of breath the same, better or worse today?”).

**Impact**

- The clinicians expressed a perceived impact on their patients in quality of life and self-management and confidence.

## Discussion

This TEC4Home feasibility study, a precursor to the full TEC4Home RCT to follow, focused on exploring 3 key issues: (1) Would the study procedures be acceptable to both patients and providers? (2) Would the evaluation framework capture useful metrics for outcome evaluation? (3) Did the results suggest a trend toward HHM improving the care of patients with HF?

### Insights From the Results of the Feasibility Study

The purpose and scope of this feasibility study were not designed to determine the efficacy of HHM. Nevertheless, we observed important trending of the data showing benefits across all 3 domains of the Triple Aim Plus framework. Most importantly, fewer patients had unscheduled ED revisits, fewer were readmitted to hospital, and the overall length of hospitalization decreased. In terms of quality of life, participants reported improved scores for both HF and general questionnaires. Furthermore, in economic terms, a cost consequence analysis showed that in all health care utilization factors measured, TEC4Home participants demonstrated decreased cost to the health system and decreased out-of-pocket costs. Finally, both participants and providers felt an improved experience in managing the patient’s HF.

The observation that our participants felt better while being monitored was similar to clinical case series and studies demonstrating high participant satisfaction when patients with HF were monitored at home [16]. Critically, our positive results differ from those of previous studies that suggested no benefit [3,4]. We hypothesize that TEC4Home was designed to support a patient in the postdischarge period for 60 days, which may be a reason for our promising results. Our study also measured participants’ quality of life and end-user experience, and both were found to increase. These findings have not been frequently reported in the literature.

### Acceptability of the Conduct of Research

This study, conducted in 3 urban sites in British Columbia, enrolled motivated patients. Of the 219 patients fulfilling eligibility criteria, only 70 patients enrolled, resulting in a recruitment rate of 32.0%. It would, therefore, be important to increase identification, recruitment, and enrollment of eligible patients. One challenge experienced was attempting to recruit patients at the height of their exacerbation in the ED. Indeed, other studies have encountered similar challenges in recruiting from the ED with common difficulties, such as time-consuming health record searches, limited research nurse support, and lack of face-to-face communication between patient and researcher [17]. The results we provide in Table 1 helped us to understand some of the factors that led to eligible patients declining participation, such as not feeling well enough to participate and not feeling comfortable using HHM or having someone to help them use it.

We presented these findings to our research study patient advisory committee and collectively developed the following ways to improve recruitment of eligible patients: increase engagement with the hospital staff members to raise awareness of TEC4Home to improve referral rates of potential patients, and identify optimal times to approach patients during patients’ hospital stays (eg, when they begin to feel better) or being careful to avoid recruiting while in the ED, especially when patients are being admitted to hospital.

The feasibility study also helped us to devise the following refinements to recruitment procedures: refining recruiter materials, including brochures, videos on how to use the HHM tool kit, and other materials to demystify study procedures and pique the participants’ interest; reviewing and clarifying the eligibility criteria with recruiters so they can conduct the screening and enrolment of patients more effectively; and establishing an ongoing support-and-feedback loop for site recruiters to promote consistent understanding and support for patient engagement.

Although a before–after analysis using administrative data is appropriate for a feasibility study, a more definitive trial design with a true control comparison is required. One site was unable to provide administrative data. Further, the discrepancy in responses and return rates between administrative data and survey data will be addressed in the RCT with an emphasis on provincial databases being the definitive source for our primary outcome.

Our results suggests that participants are generally able to perform self-monitoring routinely over 2 months postdischarge from the hospital. Our participants submitted data on nearly 95% of days, which compares favorably to similar studies. A recent telemonitoring feasibility study with patients with type 2 diabetes mellitus found lower adherence rates compared to our trial [18]. We further found the success rate of presurvey completion to be 67% (47/70), postsurvey completion to be 70% (49/70), and pre- and postsurvey completion by the same participants to be 50% (35/70). We identified ways, such as using scheduled reminders, to increase survey returns in our RCT.

Comments from patients in the survey revealed that those who persisted in using the HHM found the experience very helpful to support their self-management. Technology usability was another area that needed improvement—how to make the equipment more user friendly. Survey comments from monitoring clinicians were also highly useful to improving the types of data we needed to include in the dashboard, the workflow of monitoring nurses when contacting participants and their PCP, and how often the monitoring report summary should be sent to PCPs. We were unable to obtain feedback from participants’ PCPs in this feasibility study and needed to understand the reasons for it. We conducted a focus group with the PCPs to explore this issue prior to conducting the full RCT.

### Evaluation Framework

This study allowed us to examine our data collection procedures and scope. Overall, our evaluation framework was able to guide the collection of metrics to assess the outcomes that we would like to measure: patient outcome, end-user experiences, and health system cost-effectiveness. The data we collected helped us determine the primary outcome differences before and after monitoring, a basis for hospital utilization and cost comparisons, and end-user experiences based on the validated scales that we selected. This study also guided us in improving the scope of data we would be collecting, such as additional provincial databases covering clinical baseline measures to learn more about our participants at enrollment (eg, severity of illness, comorbidities), more health care utilization indicators (eg, specialized medical services), health care system and patient costs (eg, prescription drugs dispensed), and vital statistics that include accurate and more detailed mortality data. Additionally, the lack of data from a control comparison is a true limitation of any feasibility study design and will be addressed in the upcoming RCT. We also lengthened the period of data collection from just 90 days before and after enrollment to include 3 additional 3-month periods, up to 1 year before and after enrollment to provide insight into long-term effects. A shift from the VR-12 to the EuroQol-5D (EQ-5D) quality of life assessment tool will allow for the calculation of quality-adjusted life-year analysis. All of these refinements based on the feasibility results were implemented in the second phase of TEC4Home, a large-scale RCT designed to further examine home health monitoring across 22 British Columbia hospitals from urban, regional, and rural communities.

### Conclusions

This feasibility study better prepared us for a planned multicenter RCT by helping us understand how best to engage patients in eligibility assessment, recruitment, and retention and how to refine our evaluation framework and metrics collection. Furthermore, analysis of the data we collected provided encouragement that HHM can be beneficial for patients with HF post discharge. Findings from this feasibility study provided practical lessons that allowed us to conduct the multicenter RCT as well as identify early positive signals of the benefits of HHM for patients with HF.

## Appendix

Multimedia Appendix 1 Feasibility study patient participant eligibility criteria.

Multimedia Appendix 2 Monitoring nurse interview protocol.

Multimedia Appendix 3 Primary care physician survey protocol.

Multimedia Appendix 4 Patient participant interview analysis code book.

Multimedia Appendix 5 Monitoring nurse interview analysis code book.

Multimedia Appendix 6 Summary of most common reasons (not exhaustive) for patients not participating.

Multimedia Appendix 7 Summary of reasons provided by eligible patients who declined to participate.

## Abbreviations

CHEOS: Centre for Health Evaluation & Outcome Sciences
ED: emergency department
EHFScBs-9: European Heart Failure Self-Care Behaviour Scale 9
EQ-5D: EuroQol-5D
KCCQ-12: Kansas City Cardiomyopathy Scale
HF: heart failure
HHM: home health monitoring
PCP: primary healthcare provider
RCT: randomized controlled trial
REDCap: Research Electronic Data Capture
Tec4Home: Telehealth for Emergency-Community Continuity of Care Connectivity via Home Telemonitoring
TIM-HF2: Telemedical Interventional Management in Heart Failure II
UBC: University of British Columbia
VR-12: Veterans Rand 12-Item Health Survey
