# Supplementary material for: Testing the Feasibility of Sensor-Based Home Health Monitoring (TEC4Home) to Support the Convalescence of Patients With Heart Failure: Pre–Post Study
Source: JMIR Form Res. 2021 Jun 3;5(6):e24509. doi: 10.2196/24509 (PMC8212633; doi:10.2196/24509)
Supplement: Multimedia Appendix 1 [file formative_v5i6e24509_app1.docx]

### Feasibility Study Patient Participant Eligibility Criteria

| **Target Patient Population** Patients with Heart Failure presenting to the Emergency Department (ED) or admitted to the hospital because of their Heart Failure symptoms. | |
| --- | --- |
| **Inclusion Criteria**  To be eligible for inclusion in the study, patients with Heart Failure must: | **Exclusion Criteria**  The following will make patients with Heart Failure ineligible to participate: |
| 1. Have one or more typical symptoms (i.e. dyspnoea at rest or minimal exertion (includes orthopnoea, reduced exercise tolerance)), AND 2. Have one or more typical signs (i.e. elevated jugular venous pressure, pulmonary crepitations, pleural effusions, peripheral oedema), AND 3. Have one or more objective measures of heart failure:  - Radiological congestion (see note below). - Elevated BNP ≥ 400 pg/mL or NTproBNP ≥ 1000 pg/mL. - Reduced left ventricular ejection fraction <40% (or <45%) in previous 12 months. - Diastolic dysfunction including tissue Doppler E/e' ratio > 15 in previous 12 months. - Pulmonary capillary wedge pressure >20 mmHg.  1. Diuretic therapy. The additional value of diuretic therapy (IV or oral) is debatable, as presumably unlikely (or unsafe) that patients with genuine HF will be discharged without diuretic. 2. Be 19 years of age or older. | 1. Inability.  - Physical barriers (e.g. unable to stand on a weight scale), unless suitable caregiver support. - Cognitive impairment (e.g. MMSE <20), unless suitable caregiver support. - Unable to read and understand English, unless suitable caregiver support. - Documented history of current and active substance misuse (within 3 months) that is not well managed. - Unavailable for the 60-day monitoring period.  1. Access.  - Lack of landline or mobile phone connection to connect with the nurse. - No regular care provider e.g. GP, or at least regular walk-in clinic. - Lives outside of the home health monitoring service area.  1. Reduced potential benefit of the intervention.  - Existing intensive system of care: LVAD, transplant, dialysis. - The anticipated improvement due to revascularization (PCI/CABG) or valve intervention during the index hospitalization. - Anticipated survival < 90 days. Active palliative care, less than level III care, disseminated malignancy.  1. Is 18 years of age or younger. |

Note: Radiological congestion as an ‘objective’ measure of abnormal cardiac structure/function has significant limitations, and risks recruitment of patients without HF (e.g. overweight, breathless, swollen ankles, CXR venous changes or underpenetrated), in turn decreasing the apparent effect size of the intervention. The importance of recruiting patients with genuine HF was highlighted in the TOPCAT trial.2 Eligibility required one of two criteria 1) previous HF hospitalization 2) elevated BNP/NT-proBNP. In the overall trial, spironolactone failed to reduce the primary endpoint (CV death, aborted cardiac arrest, or HFH). However, the treatment effect was significantly different in patients recruited by the two criteria: NP HR 0.65 (0.49-0.87) versus HFH HR 1.01 (0.84-1.21).

Pragmatically radiological congestion is almost unavoidable for an ED focused study with limited immediate imaging and biomarkers. A reasonable compromise would be to 1) ensure information is collected on other criteria e.g. LVEF from charts 2) Phase I pilot send routine BNP/NT-proBNP for all patients since clinically indicated and available in St. Paul’s Hospital and Vancouver General Hospital. Subsequent phase, likely send/store baseline BNP/NT-proBNP for all patients even if result not immediately available and actionable, for quality control purposes to allow inclusion criteria alteration during the study if required.

The criteria above would include a reasonable proportion of HF with preserved ejection fraction (HFPEF) though the BNP/NT-proBNP criteria, and a smaller proportion with echocardiographic diastolic dysfunction when records are available.
