# Supplementary material for: Testing the Feasibility of Sensor-Based Home Health Monitoring (TEC4Home) to Support the Convalescence of Patients With Heart Failure: Pre–Post Study
Source: JMIR Form Res. 2021 Jun 3;5(6):e24509. doi: 10.2196/24509 (PMC8212633; doi:10.2196/24509)
Supplement: Multimedia Appendix 2 [file formative_v5i6e24509_app2.docx]

### Monitoring Nurse Interview Protocol

| **Content Area** | **Question** |
| --- | --- |
| Satisfaction | 1. Overall, are you satisfied with your experiences with the TEC4Home COPD/HF home health monitoring program? How could your satisfaction be improved? |
| Patient and Care-Provider Interactions | 1. Are you satisfied with the level of care that you were able to provide using the HHM technology? In what instances was it more successful than others? 2. What challenges did you face throughout your study involvement? What resources could have supported you in these instances? 3. Was communication with patients and patients’ family members and/or caregivers effective? How could it have been improved? 4. Aside from the patient and their family members/caregivers, who else did you communicate with during your study participation (i.e. other health professionals)? Was this communication effective, and how could it have been improved? Were these individuals responsive to the HHM intervention? 5. Can you identify any unique patient cases that stand out? What helped to increase intervention effectiveness in these situations? 6. In your opinion, does TEC4Home improve coordination of care and communication between healthcare providers and patients? How so? |
| Procedures and Usability | 1. Were all necessary procedures and protocols in place? Did any situations arise that fell outside of established protocols, and if so, how did you handle them? 2. Were there any further challenges relating to your study involvement that you would like to comment on? If so, do you have any ideas for how these may be addressed?    1. I.e. Workflow, personal, technology usability, communication |
| Impact | 1. How did this intervention impact patient’s care and experience post-discharge, following their disease exacerbation exacerbation? 2. Based on your experiences, do you have any insight and/or recommendations regarding how to best set up this position (monitoring clinician) in the future?    1. I.e. level of skill required, training, location 3. Is there anything that we have not discussed that you would like to mention related to your experiences participating with TEC4Home? |
