# Supplementary material for: Testing the Feasibility of Sensor-Based Home Health Monitoring (TEC4Home) to Support the Convalescence of Patients With Heart Failure: Pre–Post Study
Source: JMIR Form Res. 2021 Jun 3;5(6):e24509. doi: 10.2196/24509 (PMC8212633; doi:10.2196/24509)
Supplement: Multimedia Appendix 3 [file formative_v5i6e24509_app3.docx]

### Primary Care Physician Survey Protocol

**Primary Care Provider Post-Study Survey**

*Please indicate your level of agreement with the following statements.*

|  | **Strongly Agree** | **Agree** | **Neutral** | **Disagree** | **Strongly Disagree** | **N/A** |
| --- | --- | --- | --- | --- | --- | --- |
| 1. I received enough information about TEC4Home when my patient first enrolled in the program, and I clearly understood the study’s purpose, implementation, and procedures. |  |  |  |  |  |  |
| 1. I was satisfied with the overall level of communication between myself and the monitoring nurse. (If you never connected with the monitoring nurse, select “N/A”.) |  |  |  |  |  |  |
| 1. When my patient completed monitoring with TEC4Home, the final report I received from the monitoring nurses was sufficient. |  |  |  |  |  |  |
| 1. TEC4Home improved my ability to help my patient manage his/her condition from home. |  |  |  |  |  |  |
| 1. I feel that TEC4Home had a positive impact on my patient’s quality of life. |  |  |  |  |  |  |
| 1. My engagement in TEC4Home improved my outlook on home-health monitoring. |  |  |  |  |  |  |

1. You received a patient report from our monitoring nurses every two weeks. Did those reports come at a decent frequency?
   - No, the reports did not come frequently enough.
   - Yes, receiving a report every 2 weeks was sufficient.
   - No, the reports came too frequently.
   - I do not recall receiving a report / I never received a report.
2. Was the information provided in the reports:

| Informative? | - Yes | - No |
| --- | --- | --- |
| Useful? | - Yes | - No |

1. How did your patient’s participation on TEC4Home HF impact your workload?

- It decreased my workload.
- It did not affect my workload.
- It increased my workload.

1. Please provide any additional comments on how TEC4Home may have impacted patient outcomes, clinical decision making, and/or convenience or quality of healthcare delivery.
2. Is there anything that was not already discussed that you would like to mention, relating to your experiences participating with TEC4Home or any other aspect of the project?
