# Supplementary material for: Testing the Feasibility of Sensor-Based Home Health Monitoring (TEC4Home) to Support the Convalescence of Patients With Heart Failure: Pre–Post Study
Source: JMIR Form Res. 2021 Jun 3;5(6):e24509. doi: 10.2196/24509 (PMC8212633; doi:10.2196/24509)
Supplement: Multimedia Appendix 4 [file formative_v5i6e24509_app4.docx]

### Patient Participant Interview Analysis Code Book

| **Code** | **Definition** |
| --- | --- |
| Preparation and Orientiation (Feeling Informed) | The participant describes whether enough information was provided about the project and participation. The participant may make recommendations or will discuss specific aspects where more informating could/should have been provided. |
| Technology usability | The participant describes their experiences with the technology. Topics discussed may include user friendliness/ease of use, training provided, troubleshooting support/resources, reliability, etc. |
| Impact | The participant describes the overall impact of the intervention on his/her health and well-being throughout the monitoring period. The participant may discuss any behaviour changes experienced as a result of participation in the project. |
| Communication and Improving relationships | The participant describes if/how the relationship with their healthcare providers changed throughout the monitoring period. Participants might bring up communication with physicians or quality of care as areas where change occurred. |
| Quality of Life | The participant states if/how the monitoring and care plan provided by the intervention may have impacted their own quality of life |
| Project Satisfaction | The participant expresses whether he/she was satisfied with their participation in the project and whether it was a valuable experience. The participant may describe the aspects of intervention that contributed most to satisfaction, and aspects that least contributed to satisfaction |
| Sense of agency in own health | The participant describes whether or not the monitoring empowered them to become more involved in their own health and health care management. The participant may also discuss the feeling associated with this sense of being an active member in their own health management. |
| Level of Support | The participant discusses whether or not the daily monitoring and nurse contact provided a sense of support that is above than that would be expected from the usual care provided to a HF participant. The participant may also discuss the feelings associated with receiving this type of support. |
| Challenges experienced | The participant expresses any difficulties experienced throughout their participation in the project. Challenges stated can encompass the care received, research related task, technology issues, communication breakdowns, etc. |
| Recommendations | The participant provides areas of improvement or any other recommendations they may have to improve the service(or research processes) in the future |
| Other/ miscellaneous | The participant mentions insights that do not fit into any above code. |
