# Supplementary material for: Testing the Feasibility of Sensor-Based Home Health Monitoring (TEC4Home) to Support the Convalescence of Patients With Heart Failure: Pre–Post Study
Source: JMIR Form Res. 2021 Jun 3;5(6):e24509. doi: 10.2196/24509 (PMC8212633; doi:10.2196/24509)
Supplement: Multimedia Appendix 5 [file formative_v5i6e24509_app5.docx]

### Monitoring Nurse Interview Analysis Code Book

| **Code** | **Definition** |
| --- | --- |
| Project Satisfaction | The clinician describes level of satisfaction with his/her participation in the project. |
| Level of Patient Care | The clinician describes his/her satisfaction with level of care provided through the intervention. Includes successes/challenges that may have been experienced |
| Level of Support | The clinician discusses the resources he/she had access to in resolving any issues or challenges experience during the project. Resources can include from training, technical troubleshooting, care, recruitment, research, etc. |
| Communication with patients | The clinician describes how effective or ineffective the communication with patients/caregivers was with the HHM technology. Any unique cases where communication was strong or any breakdowns in communication may be presented. The clinician may also discuss who he/she was in contact with and how it benefitted the patient's care. |
| Responsiveness | The clinician discusses the responsiveness of others to his/her communication. This can include responsiveness from patients/family caregivers, patients clinical team, or technical support from the UBC or TELUS team |
| Coordination of Care | The clinician will discuss how his/her communication plan enabled or hindered the coordination of care by health professionals in the patients circle of care. |
| Quality of Life | The clinician states if/how the intervention and provision of care provided by the intervention may have impacted the patients quality of life |
| Self-care/management | The clinician alludes to any observed/percieved effect on the patient's ability to care for or manage his/her own condition |
| Protocol readiness | The clinician describes if the monitoring protocol and systems were in place to handle any unique situations/patient cases. The clinician may also suggest improvements or alternative thoughts on how to improve. |
| Challenges experienced | The clinician expresses any difficulties experienced from the provision of care, any research related task or technology issues. |
| Impact | The clinician describes the overall impact of the intervention on patient care and experience throughout the monitoring period. |
| Areas for Improvement | The clinician makes recommendations or suggests areas where the project can improve. Feedback can encompass the streams of care, research, and technology. |
| Other/ miscellaneous | The clinician mentions insights that do not fit under any other code above. |
