# Supplementary material for: Testing the Feasibility of Sensor-Based Home Health Monitoring (TEC4Home) to Support the Convalescence of Patients With Heart Failure: Pre–Post Study
Source: JMIR Form Res. 2021 Jun 3;5(6):e24509. doi: 10.2196/24509 (PMC8212633; doi:10.2196/24509)
Supplement: Multimedia Appendix 6 [file formative_v5i6e24509_app6.docx]

### Summary of most common reasons for patients not participating (*not an exhaustive list)

| **Reason for not participating** | **Number** |
| --- | --- |
| Did not meet clinical eligibility criteria | 74 patients |
| Unable to provide informed consent and no support | 55 patients |
| Resides outside of study location coverage | 24 patients |
| History of non-compliance based on medical staff assessment | 24 patients |
| Cannot use equipment and no support | 21 patients |
| Condition too unstable | 20 patients |
| Documented substance misuse in past 3 months that is not well managed and may hinder participation | 18 patients |
| Patient declines to participate | 121 patients |
