# Supplementary material for: Testing the Feasibility of Sensor-Based Home Health Monitoring (TEC4Home) to Support the Convalescence of Patients With Heart Failure: Pre–Post Study
Source: JMIR Form Res. 2021 Jun 3;5(6):e24509. doi: 10.2196/24509 (PMC8212633; doi:10.2196/24509)
Supplement: Multimedia Appendix 7 [file formative_v5i6e24509_app7.docx]

### Summary of reasons provided by eligible patients who declined to participate

| **Refusal Reason** | **Number** |
| --- | --- |
| No reason given | 58 patients |
| Other reason | 24 patients |
| Patient and/or family caregiver does not think he/she can perform study procedures | 14 patients |
| Patient and/or family caregiver does not want to add extra care | 10 patients |
| Patient does not they are suitable to participate in study | 5 patients |
| Negative perceptions of technology | 4 patients |
| Family-member and patient disagree on participation | 2 patients |
| Patient is unsure about future living conditions | 2 patients |
| Patient declined to speak to a researcher | 2 patients |
